# Hemorrhaged pineal arteriovenous malformation in an older adult patient: Case report and literature review

**DOI:** 10.1016/j.radcr.2025.08.081

**Published:** 2025-09-22

**Authors:** Shota Yoshimura, Kosuke Hirayama, Mitsuru Noda, Shiro Obata, Yoshiharu Tokunaga, Takayuki Matsuo

**Affiliations:** a Department of Neurosurgery, Nagasaki Prefecture Shimabara Hospital 7895, Shimokawajiri-machi, Shimabara, 855-0861, Japan; b Department of Radiology, Nagasaki Prefecture Shimabara Hospital 7895, Shimokawajiri-machi, Shimabara, 855-0861, Japan; c Department of Neurosurgery, Nagasaki University Graduate School of Biomedical Sciences, 1-7-1, Sakamoto, Nagasaki, 852-8501, Japan

**Keywords:** Pineal arteriovenous malformation, Flow-related aneurysm, Stereotactic radiosurgery

## Abstract

We present a case of a pineal arteriovenous malformations (AVMs) with intraventricular hemorrhage and hydrocephalus in an older adult patient. Imaging revealed a 7 mm nidus of the pineal region, fed by the posterolateral choroidal arteries (PLChA) and draining into the confluence of the sinuses. A 2 mm distal flow-related aneurysm of the PLChA is identified. Linear-accelerator-based stereotactic radiosurgery was used to treat the nidus and aneurysm. Four months later, MR angiography revealed the disappearance of the aneurysm. The patient remained rebleed-free for 4 years until death. This case features rare elements: late-onset hemorrhage, radiotherapy for flow-related aneurysms, and rapid postirradiation aneurysm resolution.

## Introduction

Arteriovenous malformations (AVMs) are characterized by congenital high-flow shunts between arteries that lack a muscularis layer and veins, with a central tangled nidus devoid of capillaries [1]. Brain AVM (BAVM) accounts for approximately 2% of intracranial hemorrhages, with an incidence rate of approximately 1 in 100,000 persons per year [2]. It is a major cause of hemorrhagic stroke, particularly in young people [2]. Pineal AVMs are particularly rare lesions, occurring in 1%-7% of all reported cases [3]. Moreover, patients with AVM and intracranial aneurysms reportedly experience a high incidence of both initial and subsequent rebleeding [4]. Furthermore, radiation at the nidus, including flow-related aneurysms, is uncommon, although the treatment for patients with AVM and these aneurysms has not yet been established [4].

In this report, we present an extremely rare case of a pineal AVM at the age of 80 years with intraventricular hemorrhage in which stereotactic radiotherapy was administered to the nidus, including a flow-related aneurysm.

### Case report

**Fig. 1:**
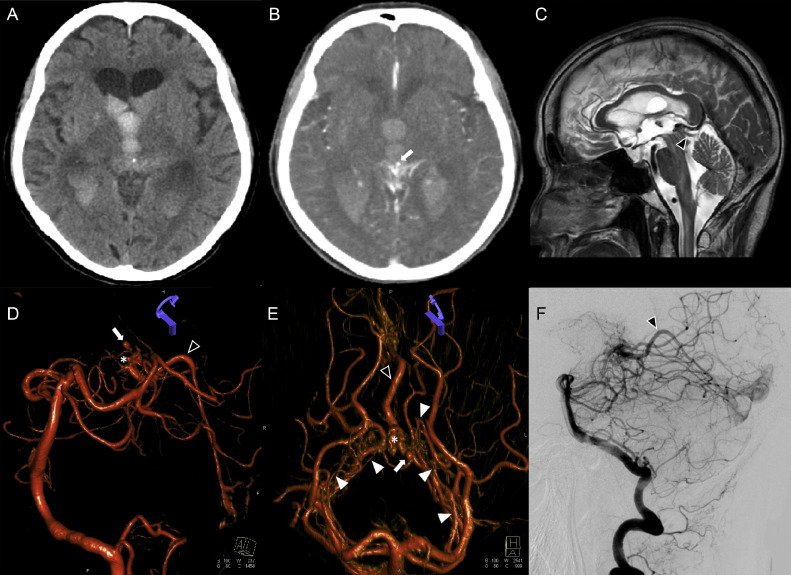
(A) Computed tomography revealed intraventricular hemorrhage and hydrocephalus. (B) Computed tomography angiography revealed an aneurysm in the region of the posterior third ventricle. (C) Sagittal T2-weighted magnetic resonance image demonstrates a flow void around the pineal body draining into the confluence of sinuses (black arrowhead). (D) Lateral view on digital subtraction angiography reveals arteriovenous malformation of the pineal region with a small nidus (asterisk), a 2 mm distal flow-related aneurysm (white arrow), and dilated veins draining into the confluence of sinuses (black arrowhead) (E) Anterior-posterior view on digital subtraction angiography reveals arteriovenous malformation of the pineal region with a small nidus (asterisk) fed by the posterolateral choroidal artery (white arrowhead) and dilated veins draining into the confluence of sinuses (black arrowhead). (F) Lateral view on digital subtraction angiography reveals dilated veins draining into the confluence of sinuses (black arrowhead).

An 80-year-old woman with a history of gastric cancer and an asymptomatic putamen hemorrhage presented to the emergency department due to a sudden onset of confusion. The patient had no family history of stroke. Upon her arrival at the hospital, her Glasgow Coma Scale (GCS) score was 11 (E1V4M6). Her pupils were both 2 mm in diameter with absent light reflexes. Manual muscle test score indicated grade 2/5 in the upper limbs and grade 3/5 in the lower limbs. Laboratory tests were within normal limits except for D-dimer at 5.2 μg/dL (normal: <1 μg/dL). A non contrast computed topmography revealed an intraventricular hemorrhage and hydrocephalus (Fig. 1A). Computed tomography angiography revealed a cluster of blood vessels in the posterior third ventricle (Fig. 1B). Sagittal T2-weighted magnetic resonance imaging revealed a flow void around the pineal body draining into the confluence of the sinuses (Fig. 1C). Digital subtraction angiography revealed a 7 mm nidus of an AVM of the posterior third ventricle fed by the posterolateral choroidal artery (PLChA), draining into the confluence of the sinuses (Fig. 1D, E and F). A distal flow-related aneurysm (2 mm) in the left PLChA was identified as the probable rupture (Fig. 1D and E).

**Fig. 2:**
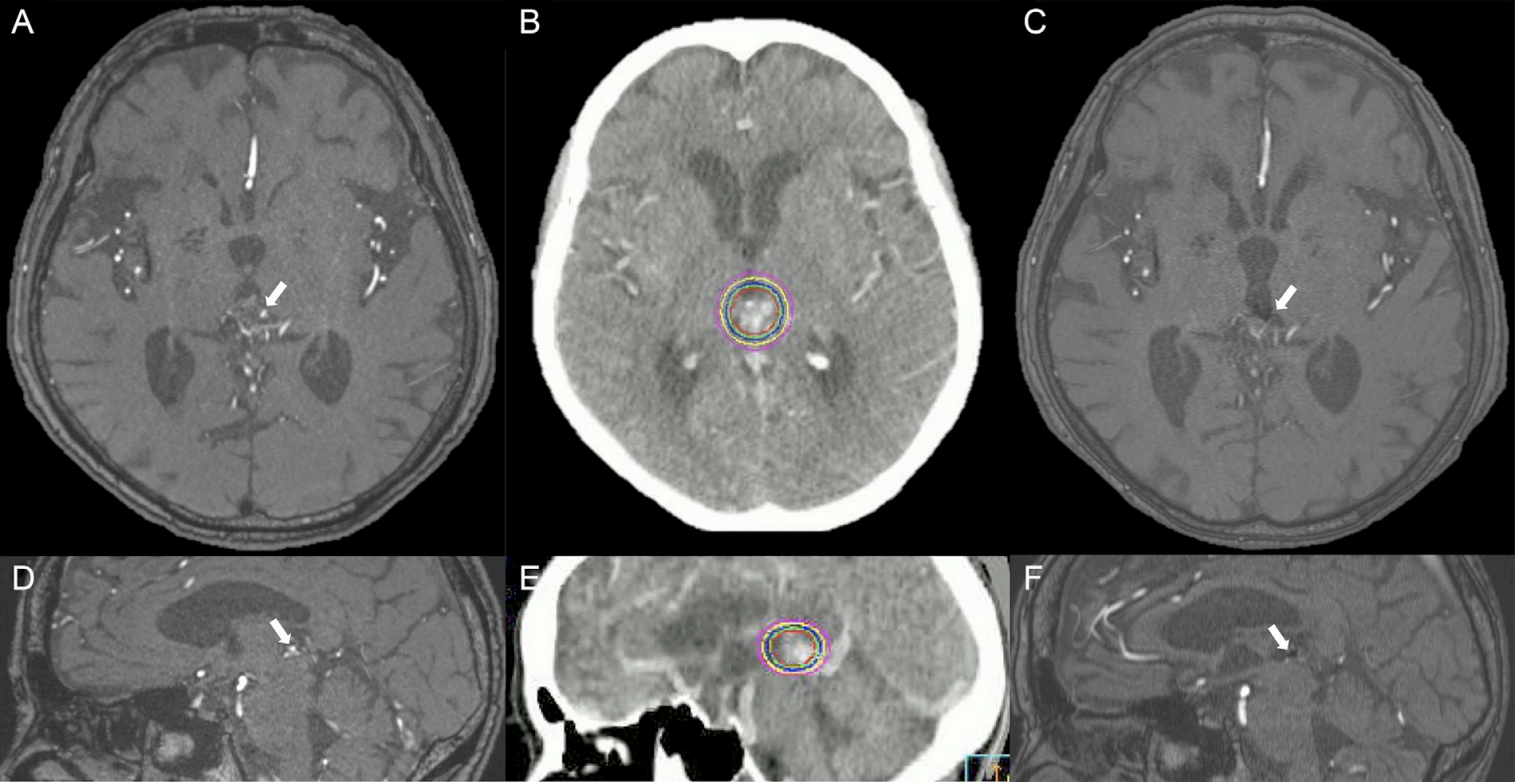
Axial (A) and sagittal (D) magnetic resonance angiography before linear accelerator-based stereotactic radiosurgery reveal an aneurysm (white arrow). Axial (B) and sagittal (E) computed tomography show the radiation field and dose color of radiation (pink circle: 12 Gy, yellow circle: 15 Gy, blue circle: 18 Gy, green circle: 19 Gy, red circle: 20 Gy). Axial (C) and sagittal (F) magnetic resonance angiography 4 months after linear accelerator-based stereotactic radiosurgery shows the disappearance of the aneurysm (white arrow).

The patient was diagnosed with Spetzler-Martin grade Ⅱ AVM. On admission, the patient underwent emergency ventricular drainage and her consciousness improved to a GCS score of 14. Due to the patient's advanced age and cognitive decline, the patient and family requested not to pursue endovascular embolization or surgical resection. One month after onset, a linear accelerator for stereotactic radiotherapy (20 Gy) in the AVM, including the flow-related aneurysms, was performed (Fig. 2B and E). The patient was transferred to a rehabilitation hospital with a modified Rankin Scale score of 2. Magnetic resonance angiography (MRA) conducted 4 months after radiotherapy revealed the disappearance of the flow-related aneurysm (Fig. 2A, C, D and F). Following this period, the patient experienced no rebleeding for 4 years and eventually died due to old age. Informed consent was obtained from the patients for publication in this journal.

## Discussion

We encountered an AVM in the pineal region with a late-onset hemorrhage. The incidence of bleeding due to AVM has been reported to be approximately 0.5 cases per 100,000 people per year, with a mean annual bleeding rate in the range of 2–4% [4]. The median age at presentation is 32 years, with 75% diagnosed before the age of 45 years and 90% before the age of 55 years and the incidence in older adults is rare [2]. The oldest age for a case of AVM hemorrhage in the pineal region was 55 years, and our patient (80 years old) breaks that record (Table 1) [[5], [6], [7], [8]]. The risk factors for hemorrhage include the presence of associated aneurysms, small size, deep venous drainage, deep location, high feeding artery pressure, and previous AVM rupture [2,9]. Our case was considered a typical high-risk case because it encompassed multiple risk factors. Patients with flow-related and intranidal aneurysms have an increased risk of bleeding, both at initial presentation and at recurrence [4]. Therefore, when BAVM resection must be postponed or stereotactic radiosurgery is performed, rapid and aggressive aneurysm management via embolization is preferred [10]. Partial embolization of flow-related or intranidal aneurysms has been reported to be useful in reducing the risk of bleeding during the latent period after stereotactic radiotherapy [4]. However, partial treatment of the nidus does not appear to have a positive impact on the natural history of the disease and may actually be harmful in some cases; there is no consensus in the literature on the best treatment strategy [4]. In the 4 previous reports of pineal AVMs presenting with hemorrhage, endovascular embolization plus resection was selected in 2 patients, resection alone in 1 patient, and radiation therapy alone in 1 patient (Table 1). The patient had a 7 mm nidus with a distal flow-related aneurysm. After endovascular embolization, surgical resection was considered. However, neither the patient nor her family wished to undergo invasive treatment because of her advanced age and dementia. Linear accelerator-based stereotactic radiosurgery, which is used in our hospital, was selected as the minimally invasive treatment method.

**Table 1:** Clinical features of hemorrhaged pineal AVM.

Author and year	Age/Sex	Presentation	Nidus size	Feeder	Drainer	Treatment	Aneurysm	Prognosis
Present study	80/ F	Intraventricular hemorrhage/ hydrocephalus	7mm	Posterolateral choroidal arteries	Draining vein into the confluence of the sinuses	RT (20Gy)	Flow related aneurysm (2mm)	No rebleeding for 4 years
Funakoshi et al. [5]	55/ M	Intraventricular hemorrhage	2cm	Bilateral PCA	Galen vein	RT (18Gy)	−	NI
Weil AG et al. [6]	50/ F	Intraventricular hemorrhage/ hydrocephalus	NI	Posterolateral choroidal arteries	Galen vein	Embolization+ Resection	Flow related aneurysm	NI
Nozaki et al. [7]	17/M	Intraparenchymal hemorrhage/intraventricular hemorrhage	3cm	Bilateral PCA,SCA,Bilateral post choroidal artery	NI	Embolization+ Resection	−	NI
Yasargil et al. [8]	46/ M	Subarachnoid hemorrhage	small	NI	NI	Resection	NI	NI

Abbreviations: AVM, arteriovenous malformation; F, female; M, male; MRI, magnetic resonance imaging; NI, no information; PCA, posterior cerebral artery; RT, radio therapy; SCA, superior cerebellar artery

Regarding linear accelerator-based stereotactic radiosurgery, 195 patients with AVMs with a mean maximum diameter of 2.61 cm and a mean volume of 6.9 mL were analyzed, and the obliteration rates at 1, 3, 5, 7, and 10 years were 17.4, 54.9, 73.8, 78.4, and 80.5% [11]. The mean dose prescribed to patients with complete obliteration was 16.87 Gy; in patients with treatment failure, it was 16.2 Gy (*P* = .002) [11]. Regarding AVM rupture status, the obliteration rate in ruptured AVMs was 89.66% and in the nonruptured AVMs was 74.07% (*P* = .006) [11]. They reported that nidus diameter and deep venous drainage were risk factors for lower obliteration rates and that preoperative embolization did not affect obliteration rates. Post-treatment hemorrhage was 8.72%, for which nidus diameter was a risk factor [11].

In our report, the flow-related aneurysm was irradiated, and MRA performed 4 months after radiotherapy confirmed the disappearance of the aneurysm (Fig. 2A–F). The presence of a patent aneurysm was significantly associated with an increased risk of rebleeding after stereotactic radiosurgery compared with patients who were clipped or embolized. Although radiation therapy is not the standard treatment for aneurysms, gamma knife radiosurgery (GKRS) has been tested for distal aneurysms for which surgical options are unavailable. Liscak et al. [12] reported cases of bleeding distal aneurysms without an AVM by targeting a single isocenter and a peripheral dose of 24–29 Gy and confirmed aneurysm obliteration after 20–36 months. In contrast, the feeding artery was occluded as a compensatory measure. However, new neurological symptoms were not observed in 3 cases of feeder occlusion [12]. In a rabbit model, aneurysm treatment with 25 Gy at 50% isodose using GKRS resulted in 1.7% size reduction per month and 0.3% wall thickening per month [13]. In our case, the interval between postirradiation and obliteration of the aneurysms was remarkably short, and the occlusion mechanism was unknown. AVM obliteration is achieved by the proliferation and degeneration of microvessels in the capillary nidus after irradiation. AVMs are intraparenchymal structures, and the connective tissue stroma surrounding them is enriched with spindle-shaped cells and myofibroblasts. Radiosurgery causes the rapid activation of myofibroblasts in the connective tissue stroma, helps the hyalinization of the occluded vessel periphery, and stabilizes AVM occlusion. However, aneurysms lack these structures and depend on slowly progressive intraluminal thrombosis for effective radiosurgery.

After successful surgical removal, histopathological examination is needed to determine whether the pineal AVM has an intraparenchymal structure. Only 1 patient with an AVM compact nidus within the pineal parenchyma has been histopathologically confirmed [10]. Similar to previous reports, the nidus of our case was as small as 7 mm, indicating that it may have been located within the pineal gland. However, a histopathological examination was not performed.

Treatment strategies for AVM include microsurgery, endovascular therapy, stereotactic radiotherapy, or an appropriate combination of these methods to ensure optimal and safe treatment. Our report highlights that radiation therapy alone might be effective for treating compact nidus AVM with distal flow-related aneurysm in older adult patients, although more cases need to be gathered for further validation.

## Conclusions

AVMs of the pineal region are rare, with very few reported cases. Our case is particular because it has several rare clinical features: late-onset hemorrhage, radiotherapy including a flow-related aneurysm, and disappearance of the aneurysm 4 months after irradiation.

## Statements and declarations

This study was conducted in accordance with the tenets of the Declaration of Helsinki.

## Patient consent

Informed consent was obtained from the patients for publication in this journal.
